# Integrating geographic information systems into veterinary education within the one health framework: an interdisciplinary approach

**DOI:** 10.3389/fvets.2025.1612524

**Published:** 2025-07-09

**Authors:** Antonio Contreras, Víctor Cuevas, Jorge Rivera-Gomis, Antonio Sánchez, Pelayo Acevedo, Joaquin Vicente

**Affiliations:** ^1^Departamento de Sanidad Animal, Universidad de Murcia, Murcia, Spain; ^2^Grupo Sanidad y Biotecnología (SaBio), Instituto de Investigación en Recursos Cinegéticos (IREC), UCLM-CSIC-JCCM, Ciudad Real, Spain; ^3^Centre for Epidemiology and Planetary Health (CEPH), Scotland’s Rural College (SRUC), Inverness, United Kingdom

**Keywords:** teaching innovation, epi map, QGIS, ArcGIS story maps, European rabies bulletin, coinfame dataset, animal tuberculosis, one health

## Abstract

**Introduction:**

Geographic Information Systems (GIS) have become essential tools in animal and public health, particularly within the One Health framework. Despite their relevance in health and environmental science programs, GIS training is not yet fully integrated into veterinary curricula. This gap limits the ability of veterinary students to effectively utilize spatial data in their future professional practice.

**Methods:**

To address this educational shortfall, we implemented a teaching innovation project at the University of Murcia, aimed at incorporating GIS training into veterinary education. Using open-access tools, we designed a series of tutorials, manuals, and exercises with graduated levels of complexity. These resources were based on real and simulated epidemiological datasets and focused on topics relevant to animal and public health. The tools employed included online GIS platforms such as the European Rabies Bulletin (ERB), free desktop applications like Epi Map from Epi Info 7 and QGIS, as well as ArcGIS Online, including its story map functionalities.

**Results:**

The educational pathway developed introduces GIS through a stepwise progression: (1) use of the ERB to explore official rabies data in Europe since 1977; (2) application of fictional disease data in rabbits using Epi Map and QGIS for beginners; and (3) analysis of official bovine tuberculosis sanitation campaigns in Ciudad Real (2007–2011) for more advanced users. The exercises support data visualization, geospatial analysis, and the generation of online outputs. They were designed to be accessible to users with no prior GIS experience and can be implemented remotely and asynchronously.

**Discussion:**

This approach offers a scalable and accessible model for integrating GIS into veterinary education. The ability to visualize epidemiological patterns and combine health and production data with environmental risk layers fosters student engagement and spatial reasoning. Moreover, the capacity to publish results online enhances dissemination and interdisciplinary collaboration. Future work will focus on evaluating student learning outcomes and refining the pedagogical design based on user feedback.

## 1 Introduction

Spatial epidemiology involves the description and analysis of geographic variations in disease distribution, considering a wide range of factors such as demographic, environmental, behavioral, socio-economic, genetic, and infectious determinants, as defined by Elliot et al. (1). In recent years, the need for and application of spatial analyses has expanded within the field of veterinary science. Advances in tools and methodologies have enhanced the discipline’s capacity to support both descriptive (2) and analytical (3) studies, as well as to develop spatial models that simulate disease spread, maintenance, and persistence (4). These models have become essential tools for the prevention, management, and eradication of disease, particularly in complex settings where pathogens circulate across multiple interfaces (5). The development of Geographic Information Systems (GIS) and different spatial and spatio-temporal statistical techniques have promoted spatial epidemiology as a relevant scientific discipline to gain a better understanding of spatial patterns, their determining factors and, above all, the development of surveillance and prevention programs and, wherever appropriate, the control of diseases (6).

GIS integrate spatial data from diverse sources, which enables efficient storage, manipulation, visualization and analysis. In recent decades, significant technological advances have enhanced the speed and accuracy of spatial data collection and management, and GIS have been established as indispensable tools across various disciplines. In the veterinary epidemiology field, GIS are particularly valuable for disease surveillance and control, as are web-based GIS support veterinary services by contributing to the management of both epidemic and non epidemic emergencies. This has facilitated epidemiological research (e.g., risk factor analysis) by evaluating disease control plans and identifying emerging gaps and challenges (7).

Veterinary students tend to be less proficient in using such tools compared to students in disciplines more closely aligned with engineering or environmental sciences, where these tools are commonly integrated into academic training. This limitation is not unique to veterinary students; similar gaps have been observed in other academic fields, such as the social sciences and education (8). Furthermore, GIS’s ability to facilitate the understanding of territorial concepts through experiential, non-memoristic learning, while simultaneously fostering the development of spatial thinking, highlights the importance of incorporating GIS into teaching and learning activities, even at the pre-university level (9). On the other hand, access to real-world databases containing disease-related information is an essential educational requirement in veterinary sciences. Despite limitations arising from personal data protection regulations, the availability of open platforms providing disease and geographic data is steadily increasing. These resources offer valuable educational opportunities for training veterinary students as well as those in related disciplines. Recently, the importance of creating these educational materials (sets of tasks) has been highlighted, not only for students but also for teachers across various fields. These materials aim to focus on working with different types of GIS technologies and provide examples of tasks that educators could immediately apply in their teaching, without the need for extensive training (10).

The GIS skills of veterinary students represent a competitive disadvantage compared to graduates from other disciplines with whom they may converge in the job market and integrate multidisciplinary professional groups. In the public health field, it is also evident that medical students lack specialized competencies in GIS, which they typically acquire through postgraduate training, particularly in epidemiology. The management of diseases at the population level necessitates the understanding and use of GIS, a requirement that increases for diseases shared among different hosts, including zoonoses. This need is most pronounced in diseases with high environmental interactions, such as those affecting extensively managed livestock populations or involving wildlife and the environment, and those in pandemic scenarios (7, 11–13). For these reasons, both animal health and public health professionals will require these competencies.

In the management of wildlife populations, professionals from environmental engineering backgrounds are often involved and typically possess solid expertise in GIS. In contrast, veterinary students frequently have limited exposure to these tools, which can hinder their ability to apply them effectively in practical contexts. Commonly used GIS platforms such as ArcGIS (14, 15)—the latter being free and open-source—require specific technical skills that are not usually included in veterinary curricula, making hands-on use difficult without prior training. Alternatively, more accessible options are available for students without prior GIS experience. Some such options that allow them to perform tasks that introduce them to the management and application of GIS are Epi Map from Epi Info 7 (16) or the Maps feature from the European Rabies Bulletin (ERB) database (17). Epi Info is an open-access statistical software widely recognized in epidemiology. It was developed by the Centers for Disease Control and Prevention (CDC, USA) with support from the World Health Organization (WHO). The ERB is managed by the WHO Collaborating Centre for Rabies Surveillance and Research at the Friedrich-Loeffler Institute (Germany). It compiles quarterly reports on rabies cases from 44 countries dating back to 1977. Although both applications are more limited than the above-mentioned GIS programs, their easy use makes them excellent educational tools, particularly in the epidemiology and animal or public health fields. Nevertheless, more complex GIS projects created with QGIS or ArcGIS and published online, or through self-executing files (i.e., html), including narrative information (story map), can be employed for demonstration and complementary formation purposes. Table 1 summarizes the ain educational objectives to be achieved.

**Table 1:** Main characteristics of the geographic information systems used and their main educational objectives.

GIS	Main Strengths	Main Limitations	Main Educational Objectives
European Rabies Bulletin (ERB) Online Open Access	User-friendly interface Integrated datasets with official WHO data Ready-to-use tools for basic map-based epidemiological analysis	Limited customization options for map design No support for high-quality image export Inability to integrate external datasets Layers cannot be modified or reconfigured Results can only be saved as static images	Creating and displaying point (symbol) maps Visualizing rabies cases by country and host Assessing the role of wild reservoirs (foxes) Evaluating the role of other wild and domestic hosts Analyzing the relevance of human and animal rabies
EpiMap (Epi Info 7) Software Open Access	User-friendly GIS software with straightforward instructions suitable for beginners Supports loading base layers such as world maps (WorldStreetMap) and vector files (SHP, KML) Allows integration of different databases Enables reconfiguration of layers after loading Permits saving project results for future use Supports exporting results as high-quality graphic	Fewer functionalities compared to other GIS Restricted to basic data visualization Lacks tools for advanced spatial analysis Does not support heatmap generation No capability for publishing interactive maps online Uncertainty regarding long-term maintenance and technical support (beyond September 30 (25))	Creating and displaying epidemiological maps, including point (symbol) and choropleth maps Visualizing spatial clustering of cases Tracking the temporal progression of the disease
Quantum GIS (QGIS) Software Open Access	Includes all previously described features Open-source system continuously updated Supports advanced spatial analyses, including geoprocessing tools Enables creation of heatmaps Allows online publication of interactive maps	Not suitable for educational use without prior GIS training Free server for online map publication up to 50 MB Limited functionality for creating and customizing story maps	Identifying spatial clusters of cases Tracking changes in disease distribution over time Comparing student-generated outputs from Epi Map with QGIS Online publications using the same dataset
ArcGIS Private Software	Includes all previously described features Highly optimized tools for creating story maps Allows online publication of maps without the storage limitations of QGIS	Not suitable for educational use without prior skills A paid license is required	Enables comparison of Epi Map and ArcGIS Online outputs using the same dataset Enhances project understanding through integration with story map presentations

Since 2021, we have incorporated GIS into university teaching through the subject “Epidemiology, Zoonoses, and Public Health” (EZPH), offered at the Veterinary School of the University of Murcia (Spain). Building on this experience, we plan to extend the initiative to other subjects within the Veterinary Medicine degree, as well as to programs in related health sciences. As part of this effort, we aim to develop interdisciplinary educational resources suitable for hybrid or fully online learning environments, to complement the flipped classroom methodology already implemented in the EZPH course. This approach involves the use of various programs—such as Epi Info, Maps from ERB, and online tools from QGIS and ArcGIS Pro—working with both simulated and real-world data on zoonotic and public health-related infections, including animal rabies and tuberculosis (TB). These resources will be made available through open-access platforms and will include not only databases and vector files for map generation but also written materials and instructional videos containing theoretical content and guided exercises. Together, these materials will provide students with opportunities to interact with GIS tools in a practical, applied context.

In this work, we present the study of geographic variability in disease using a range of examples that illustrate contrasting scenarios, research questions, and data types—including the presence of infectious diseases, which are shared at the domestic-wildlife interface, such as tuberculosis. These examples serve a didactic purpose by highlighting key factors that shape spatial patterns of disease. Such factors can be analyzed through spatial epidemiology, often in combination with other disciplines such as ecology or molecular epidemiology, using GIS as a common analytical tool.

The overall study objective is to introduce undergraduate students with no prior training in GIS to the use of these technologies in the One Health context utilizing user-friendly tools and producing results that are accessible online. This is achieved by: (1) developing maps with the European Rabies Bulletin tool to further didactically enhance spatial data representation; (2) generating the necessary databases and tutorials for developing health map visualization projects using Epi Map, QGIS and ArcGIS Online; (3) developing projects using more advanced GIS systems, which are made available to interested users as interactive, and allows them to compare and validate their results.

## 2 Materials and methods

### 2.1 Epidemiological maps of animal and human rabies cases in Europe

In order to visualize the rabies epidemiology in Europe, we utilized the Maps feature of the WHO European Rabies Bulletin (ERB). This database provides access to quarterly reports on rabies cases from 44 countries, with data categorized into four levels of administrative divisions (Nomenclature of Units for Territorial Statistics, NUTS), including groups of regions (NUTS1), regions (NUTS2) and provinces or counties (NUTS3). It includes cases involving 23 different hosts by distinguishing between imported cases and cases originating in Europe. It facilitates the evaluation of oral vaccination campaigns for foxes whose aim is rabies control in Europe (17). Using this source, we designed a series of practical exercises for veterinary students. These included the creation of epidemiological point maps to visualize reported rabies cases in European countries, covering humans, domestic animals, wildlife, and bats. Additional exercises allowed students to construct and analyze simulated epidemiological scenarios across different European contexts. To support these activities, we provided a text file and an instructional video with step-by-step guidance (Table 2).

**Table 2:** Structure of the databases generated for the development of GIS projects, including the name and characteristics of the variables and the employed coordinate reference system.

Dataset characteristics	Coinfame (a Fictitious Rabbit Disease) in Murcia, Spain			Animal Tuberculosis in Ciudad Real, Spain	
		(coinfame_map.xls)		(bov_tb_cr.csv)	
Datasets			“Cases_farms” and	bov_tb_cr.csv	
		“Dat_epidemiol_munic”	“Cases_munic”^(1)^		
Records		45	129	3,519	
Variables		10	3	16	
	Text	Cod_munic	Name_munic	Municipality	Production
	Numerical	Outbreaks	% Prevalence	Year	Sample
		Kits_born_dead/litter	Weaned_kits/litter	N_Positives	Total _Positives
		Does_dead/farm	Kits/litter	Incidence_Year	
		% Abortion	Does/farm	R_Times	Incidence_Rate
	Date	Onset_outbreak		Date	
	Coordinates	Latitude	Longitude	UTM X	UTM Y
	Alfanumeric			Code_Farm	
	Binary			Positive	Status-A
	Ordinal			Status-B	
CRS^(2)^	ETRS89/UTM Zone 30N (EPSG:25830)				

(1) Both databases only differ in the coordinate ubication (farm or municipal administration, town hall).

(2) Coordinate reference system used for both GIS projects.

### 2.2 Epidemiological maps of coinfame (fictitious rabbit disease in Murcia, southeastern Spain)

As an initial task for veterinary students in developing epidemiological maps, we used Epi Map from Epi Info™ version 7.2.4., and a fictitious disease purportedly affecting rabbit farms in the Murcia Region, southeastern Spain. We adapted the porcine disease “Infame” database (18) and we generated a new database (“coinfame_map.xls”) to represent a hypothetical rabbit disease outbreak in Murcia, Spain, by adapting the epidemiological variables from the “Infame” database (Table 2). Thus, simulated dates of case occurrences on farms, along with hypothetical farm coordinates, were incorporated into the new database. It was assumed that the suspected disease outbreaks began on September 4 (15), and continued until October 22, 2024. The points indicating case occurrences were generated from a vector layer of points created in QGIS which, albeit fictitious, follow a pattern related to the actual distribution of rabbit farms in the Murcia Region. In parallel, with the coinfame database we obtained different maps options in a QGIS project (QGIS 3.40 Bratislava).

### 2.3 Epidemiological maps of animal TB in the Ciudad Real Province (Central Spain)

The TB data pertain to cases recorded on 751 cattle farms in the Ciudad Real province (CR), Spain, between 2007 and 2011, covering beef cattle (669 farms), dairy cattle (55 farms) and fighting bulls (22 farms), obtained from livestock health campaigns conducted in CR. In addition to the number of sampled animals and the number of TB-positive results, geographical coordinates and other epidemiological indicators, such as farms’ TB status over the last 5 years, categorized as absence, occurrence, incidence or recurrence following these definitions:

Occurrence was defined as whether a farm was declared TB-positive (at least one animal tested positive for TB using the single intradermal tuberculin test, SITT) in any of the 5 years of study. Farms were coded as 1 (occurrence) or 0 (non-occurrence).

Absence referred to farms in which no animals tested positive for TB by SITT throughout the entire study period. These were coded as 1 (absence) or 0 (non-absence).

Incidence was defined as the detection of at least one SITT-positive animal after a minimum of 1 year with no positive results on the farm (new infection). Farms were coded as 1 (incidence) or 0 (non-incidence).

Recurrence was defined as the occurrence of one or more SITT-positive animals on the same farm in at least two separate years within the study period. Farms were coded as 1 (recurrence) or 0 (no recurrence).

Due to the confidential nature of data, farm locations were randomly adjusted within a radius of up to 10 kilometers using the QGIS Field Calculator tool. Furthermore, original coordinates will not be made available in the repository with the other resources. To preserve the data structure and to allow exercises with this material, a separate QGIS project was developed using a completely randomized distribution of bovine farms (“random”) to enable the use of a database with coordinates.

All this information was compiled in a csv file (bov_tb_cr.csv) (Table 2) containing 3,519 records and 16 explanatory or indicative variables. A supplementary readme file includes the variable names, definitions, and the methods used to calculate various farm health status indices. Key variables include cattle production type (beef, dairy, or fighting cattle), the number of samples collected annually per farm, diagnostic test results, and their corresponding rates (Table 2). Spatial data were incorporated into a QGIS project by georeferencing each farm. Additionally, we included data on wild ungulates hunted during the 2022/2023 season, provided by ENETwild consortium et al. (19).

With ArcGIS Pro, we incorporated the same point data to generate heatmaps following ESRI’s recommended method for visualizing point density, which is particularly useful for health mapping. We applied this process to all farms and separately to only positive cases. Furthermore, all the shapefiles created in QGIS were imported into ArcGIS Pro to develop a simplified online map viewer to allow users to toggle layers on and off. A slider plugin was implemented to compare different maps, which enhances the understanding of each method and result, with explanatory pop-up windows providing further insights.

### 2.4 Vector files with polygonal boundaries, base and raster layers, and interactive maps

The shapefiles containing municipal boundaries for the Murcia and CR provinces were downloaded from the Spanish National Geographic Institute (IGN (20)). We therefore employed the shapefile (murcia_munic.shp) containing polygon boundaries of municipal limits, along with their associated files (dbf, prj, and shx formats), and a kml file with Spanish provincial boundaries (spain_provinc.kml), or a geopackage (ciudad_real_munic.gpkg). To minimize bias related to the use of municipal polygons, we discretized the CR province with a regular hexagonal grid composed of 20 km^2^ cells, a method recently employed in animal health research within the One Health framework (2). This hexagon grid was created with the vectorial tool “*Create grid*” in QGIS and was saved in the geopackage format (hex_cr.gpkg).

To transform the table into spatial data, we created point features by linking the X and Y values from the table fields to QGIS using the “*Create Points from Table*” tool. These points were then used to add information to any vector layer. For this purpose, we used the QGIS tool “*Join attributes by location (one to many option)*.” During this process, we obtain a vector ID for each coincidence between point and shape. To join all the data in one shape, we use “*Aggregate*” to join attributes by common ID and to calculate the mean of the values. Now we have the mean values of all the farms inside the chosen forms: hexagonal and municipal in our case.

The heatmap of the coinfame_map was generated from the “cases” layer reprojected to UTM EPSG:25830 using the QGIS “*Heatmap*” tool, which applies Kernel’s Nucleus Estimation Density method, with a pixel size of 25 m and a radius of 1 km. The heatmap of TB in CR was made by selecting only the positive point farms (equaling or more than 1 positive sample) and following the heatmap representation method that is given by ArcGIS Online MapViewer. The heatmap was created for positive farms, and for prevalent, incident and recurring farms.

As base layers, we utilized WorldStreetMap (Esri®), OpenStreetMap (Open Database License), Google Satellite (©2015 Google), and Positron (Carto).

### 2.5 Accessibility of the results

All the materials generated will be made accessible to students and interested people through open platforms, such as the University of Murcia’s TV channel (tv.um.es) or institutional repositories (digitum.um.es), to provide open access to the developed resources. To visualize the results using open-source tools, we employed various interactive mapping methods. With the QGIS software, we generated interactive html maps with OpenLayers through the QGIS2Web plugin. We also utilized the QGISCloud option to obtain a URL for linking with a published online map. Finally, we utilized ArcGIS Online along with two internal applications: MapViewer (MapWeb) for interacting with the final layers, and a map slides option created with Instant App to develop a story map for interpreting cartographic methods and results.

## 3 Results

### 3.1 Rabies epidemiology in Europe

**Figure 1:**
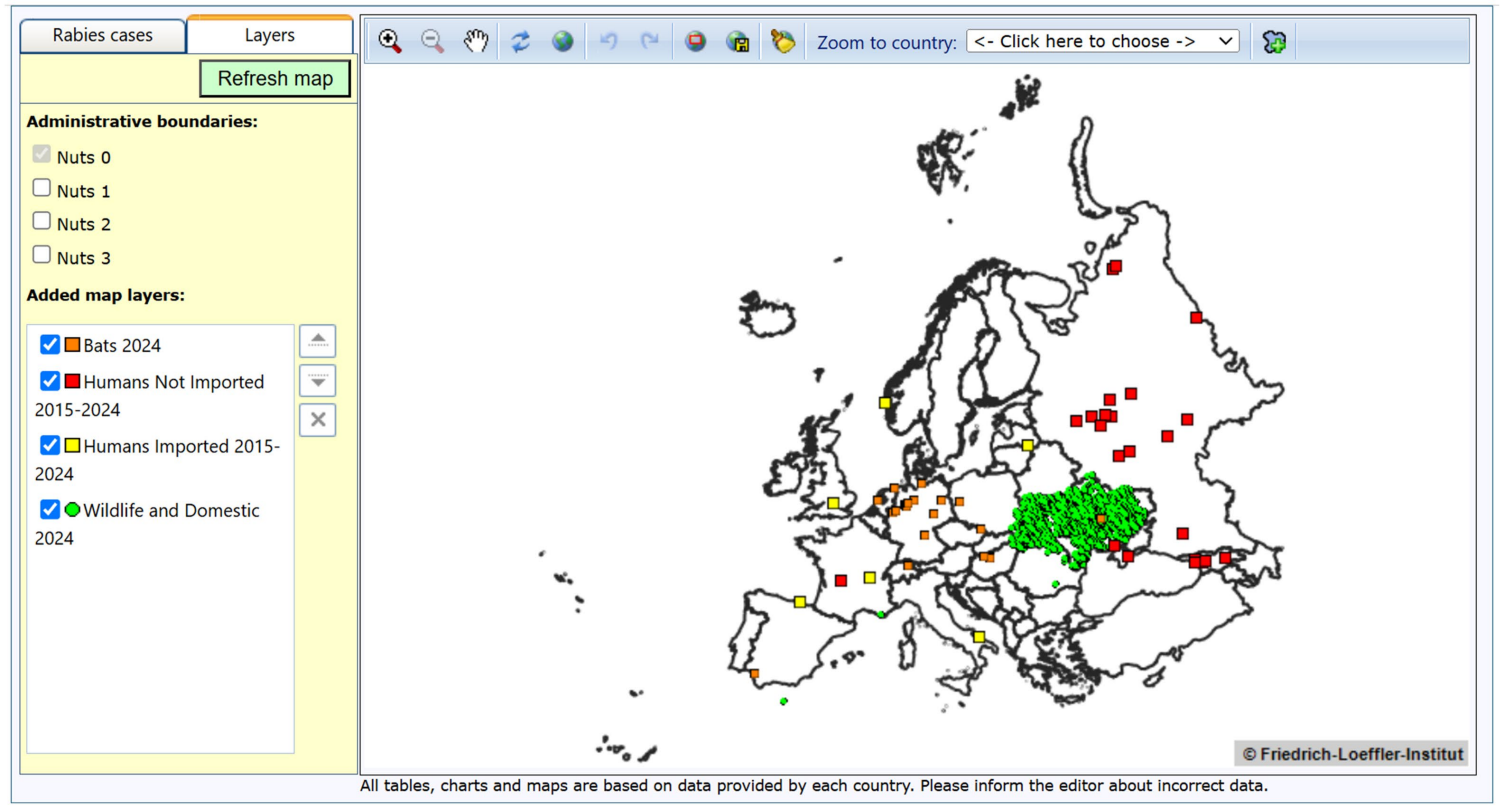
Map generated using data from the European Rabies Bulletin to summarize and update the epidemiological situation of rabies in Europe. The map illustrates reported human rabies cases in Europe over a 10-year period (2015–2024) (red squares), as well as imported human cases during the same period (yellow circles). The domestic and wild animal cases reported in 2024 are represented as green dots, while the cases reported in bats for the same year are shown as orange squares.

**Figure 2:**
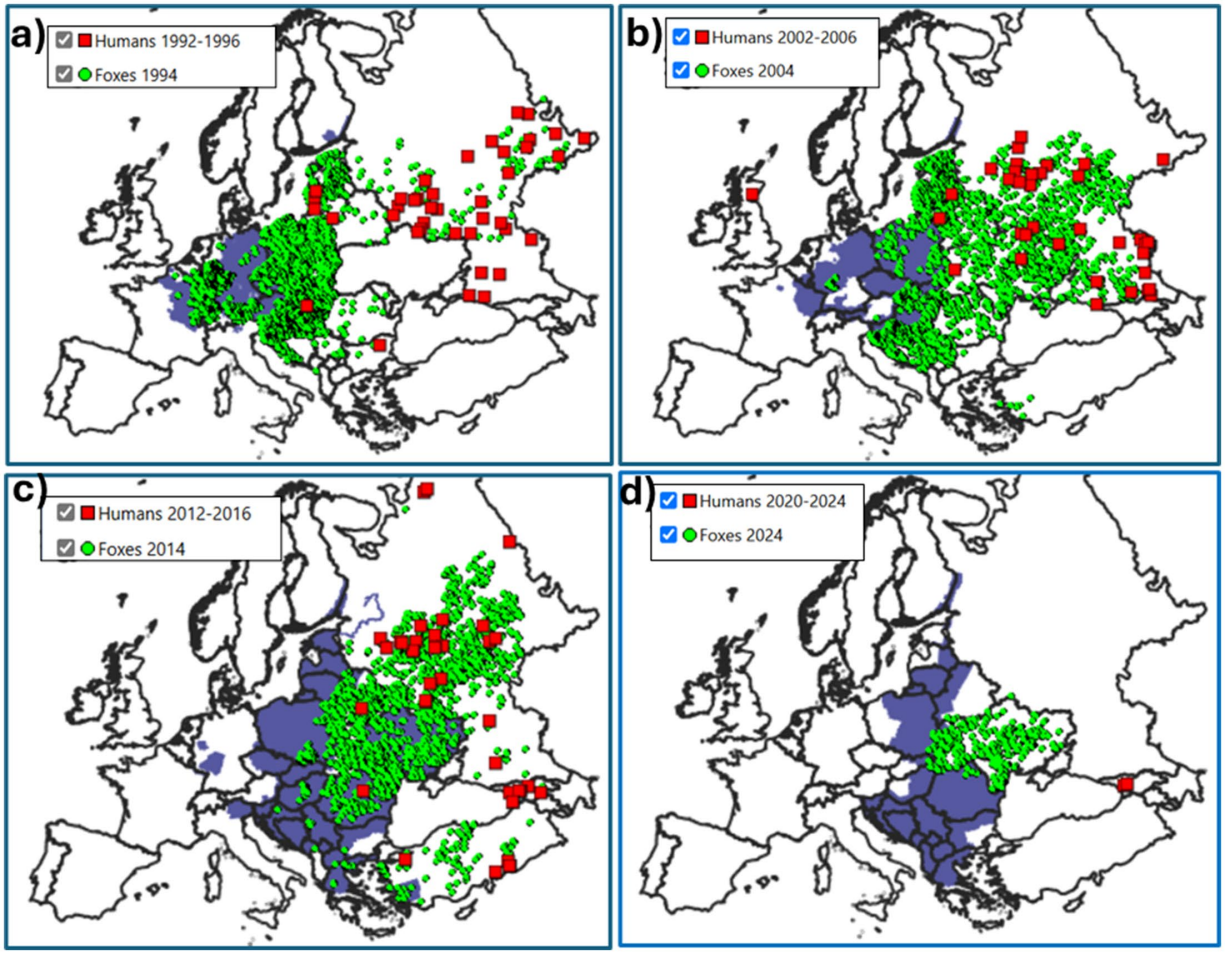
Series of maps, using data from the European Rabies Bulletin, showing rabies control strategies implemented in Europe through oral vaccination of foxes (in purple) and the evolution of reported fox and human rabies cases (green dots and red squares, respectively) over the last 40 years. The map depicting the areas where oral vaccination of foxes was implemented during 1984–1993 **(a)**; 1994–2003 **(b)**; 2004–2013 **(c),** and 2014–2015 **(d)**.

The exercises developed using data from ERB enabled students to visualize the spatial and temporal distribution of rabies cases across Europe (Figure 1). Clear patterns were observed, including the concentration of cases in specific wildlife reservoirs and a temporal decline in incidence in regions with sustained fox vaccination campaigns (Figure 2). These activities also allowed students to identify spatial mismatches between disease occurrence and vaccination coverage, which in turn prompted critical discussions during class sessions.

### 3.2 Fictitious rabbit disease in Murcia

The dataset obtained for this objective (coinfame_map.xls) contains three sheets (Table 2):

(1) dat_epidemiol_munic: it contains 45 records and 10 variables, including the municipality code and name; epidemiological data per municipality and average of the epidemiological data for farms by municipality. Most variables refer to reproductive or health parameters, such as weaned kits per litter, percent of abortion or coinfame prevalence. All the used variables, their characteristics and the Coordinate Reference System employed are detailed in Table 2.
(2) cases_farms: it contains 129 records and three variables detailing the onset date for all the 129 disease cases and the coordinates of affected rabbit farms.
(3) cases_munic: similarly to the previous file, coordinates do not correspond to the location of the suspected outbreaks, but to the municipal administration (town hall).

**Figure 3:**
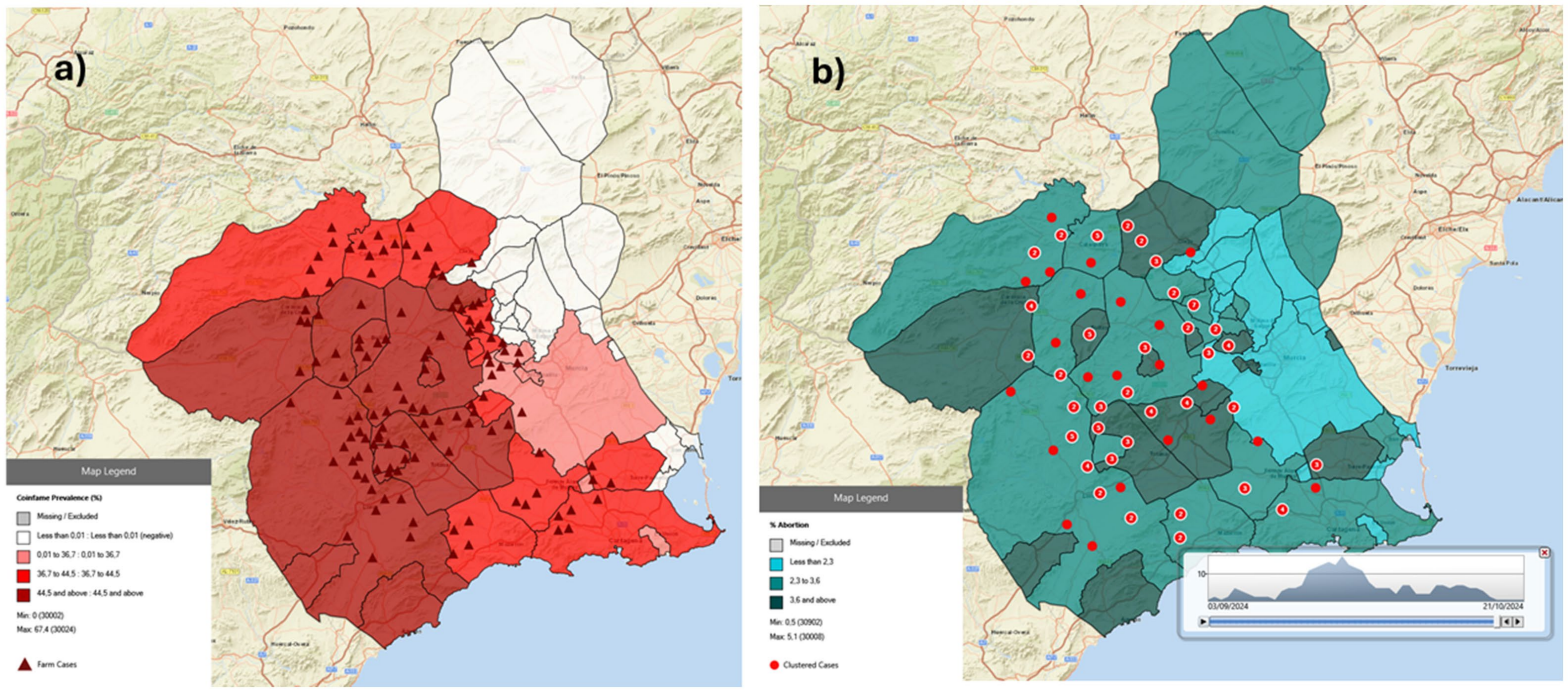
Epidemiological maps generated using Epi Map from Epi Info 7 depicting a fictitious disease (coinfame) affecting rabbit farms in the Murcia Region (Spain): **(a)** a choropleth layer representing the percentage of coinfame prevalence by municipality is overlaid with a symbol layer showing the cases on affected farms; **(b)** a choropleth layer representing the percentage of abortions on farms by municipality is overlaid with aggregated cases, including a diagram illustrating the temporal distribution of case occurrence.

Using Epi Map, we developed the following maps: choropleth maps by municipality for the quantitative variables, point (symbol) maps representing farms with cases and clustered maps of cases (Figure 3). This latter option additionally enables the visualization of temporal disease progression (“Time lapse”). All the database files necessary for generating maps are also available in the institutional repository of the Murcia University (UM). In addition, self-learning tutorials in Spanish for reproducing this project with Epi Map were also produced and organized into four practical exercises with accompanying tutorial text documents and four tutorial videos totaling 17.04 min (Table 3).

**Table 3:** Developed contents in GIS training: educational resources obtained, applications used, and open-access sources where they can be found.

Content	GIS	Geographical Scope	Resources	Format	Open Access Source	Link
Introduction to GIS for Veterinary Students			Theoretical Content	pdf	Digitum	http://hdl.handle.net/10201/150800
			Self-Assessment Files	html	Digitum	http://hdl.handle.net/10201/150902
Animal and Human Rabies Cases in Europe	European Rabies Bulletin	Europe	Exercises Tutorial	pdf	Digitum	http://hdl.handle.net/10201/146469
			Video tutorial for developing exercices (8′38″)	mp4	TV UM	https://tv.um.es/canal?serie=25395
Fictitious Rabbit Disease in Murcia (Spain)	Epi Map; QGIS	Regional (Murcia, Spain)	Dataset (Coinfame_map xls) and vector files	xls; shp; kml	Digitum	http://hdl.handle.net/10201/146363
			Exercises Tutorial	pdf	Digitum	http://hdl.handle.net/10201/146469
			Tutorial Videos (4) for developing exercises (17′04″)	mp4	TV UM	https://tv.um.es/canal?serie=25395
			Interactive map files	HTML	Digitum	http://hdl.handle.net/10201/146363
			Interactive online map	QGISCloud	www	https://shorturl.at/iQoLS
Bovine Tuberculosis in Ciudad Real (Spain)	QGIS; ArcGIS	Regional (Ciudad Real, Spain)	Dataset (bov_tb_cr.csv) and vector files	csv; gpkg	Digitum	http://hdl.handle.net/10201/153360
			Story map	ArcGIS	www	https://shorturl.at/ph27k
			Interactive story map			https://arcg.is/0OqCLO

**Figure 4:**
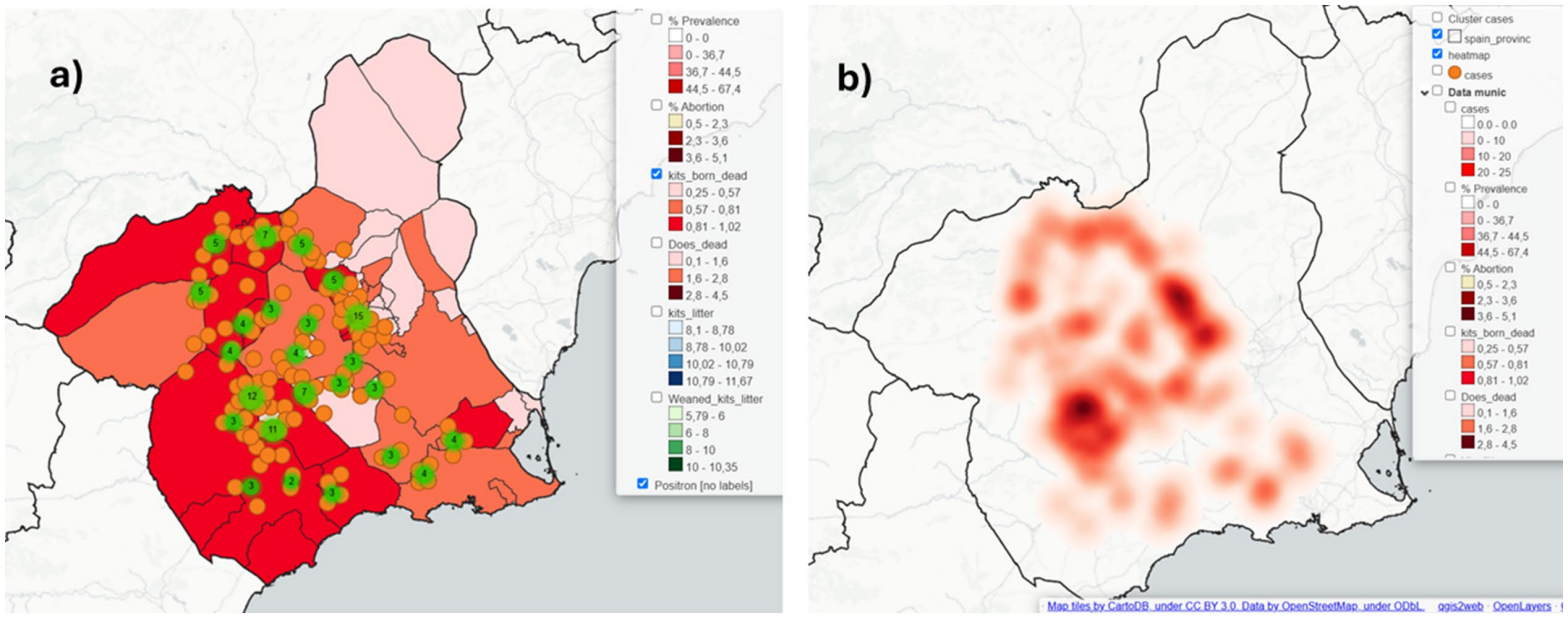
Available options for the epidemiological maps created in QGIS using the coinfame database, as presented on the interactive HTML map published with OpenLayers via QGIS2Web: **(a)** a choropleth layer displaying the mean number of stillborn kits, overlaid with two symbol layers: one representing individual cases and the other denoting clustered cases; **(b)** a heatmap generated using the coinfame case layer.

**Figure 5:**
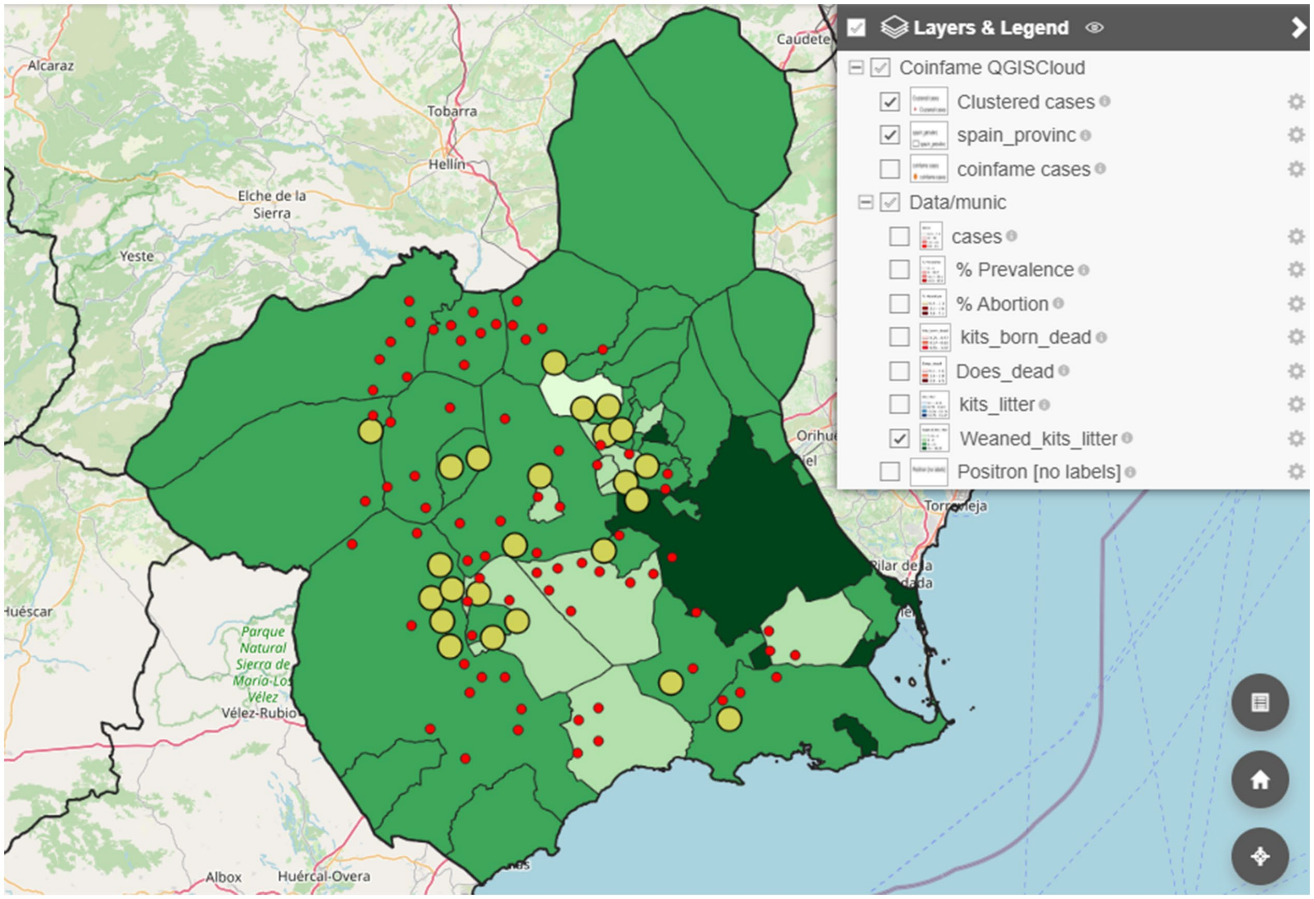
Epidemiological maps created in QGIS using the coinfame database and published online with open access via QGISCloud showing the available layers for user interactions.

Additionally, using the same datasets we developed a similar project in QGIS by incorporating advanced features, such as a heatmap of cases. The project consists of 12 layers, including three base layers and two territorial boundary layers (provincial and national). The results are accessible through an interactive html archive published using OpenLayers in QGIS (Figure 4) and via QGISCloud (Figure 5). These resources are openly available (Table 3). However, due to the memory limitations of the free QGISCloud server, the heatmap is not included in the QGISCloud online published map.

### 3.3 Epidemiological maps of animal tuberculosis in Ciudad Real (Spain)

**Figure 6:**
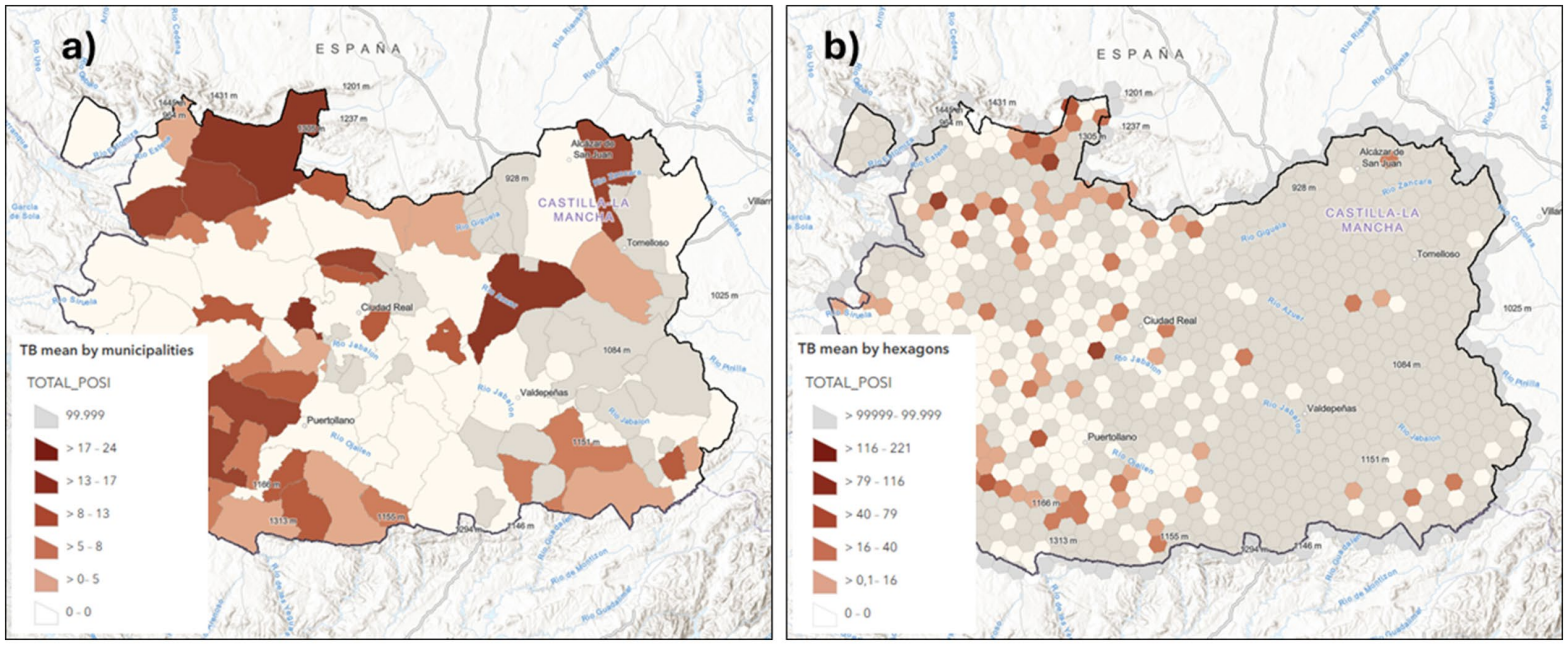
Choropleth maps showing the average total cases of cattle positive for bovine tuberculosis, represented in both municipal polygons and the 20 × 20 km^2^ hexagonal in Ciudad Real, Spain (2007–2011).

**Figure 7:**
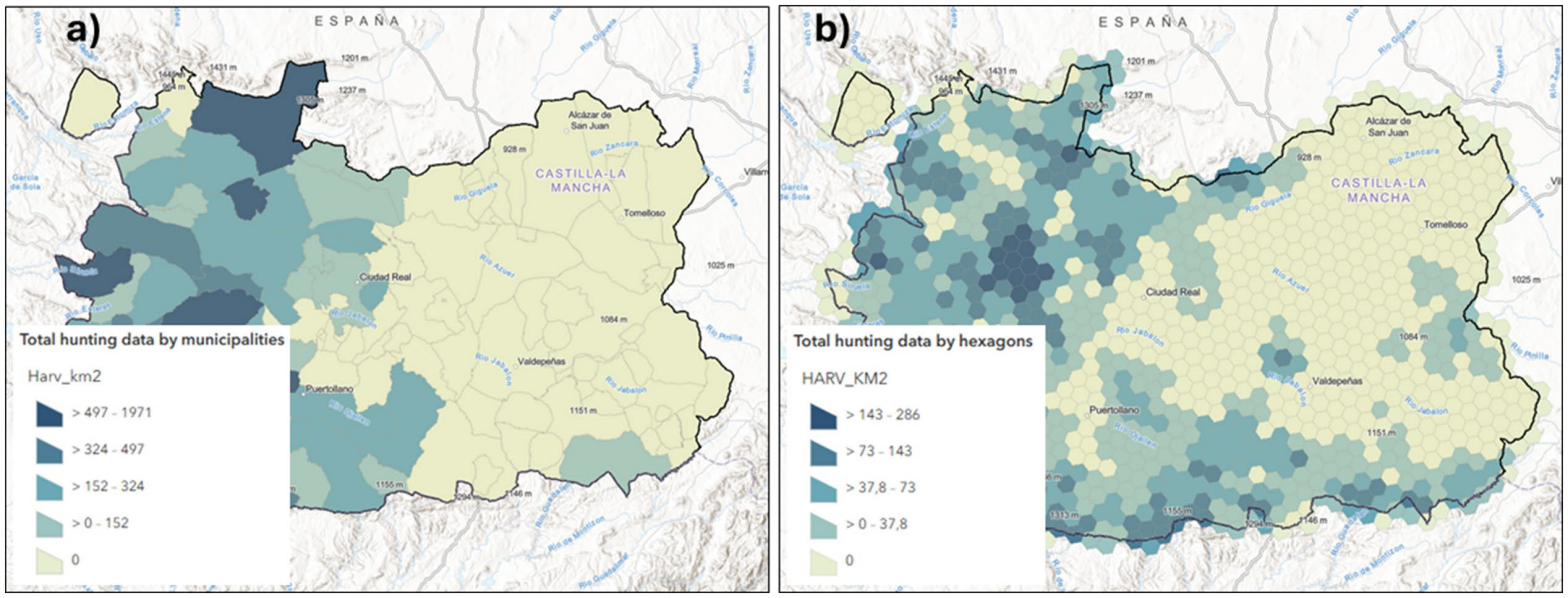
Wild ungulate harvest density layers per km^2^, displayed in municipal polygons **(a)** or in a 20×20 km^2^ hexagonal grid **(b)** in Ciudad Real, Spain (2022/2023 season).

We developed a series of maps from the aforementioned data by adding the coordinates and the values of each farm to a QGIS project: point maps to represent farms’ status, one map for the Status A variable (Free or Positive farm) and another for the Status B variable (prevalence, incidence or recurrence). We also obtained choropleth maps by adding the Status A & B point data to municipality and a regular hexagons grid (Figures 6a,b). Based on the data collected about the wild ungulates hunted during the 2022/2023 season, two layers were added to the ArcGIS project containing both the absolute data (HARVTOT) and relative data of harvest density/km^2^ (Harv_km^2^) (Figure 7).

**Figure 8:**
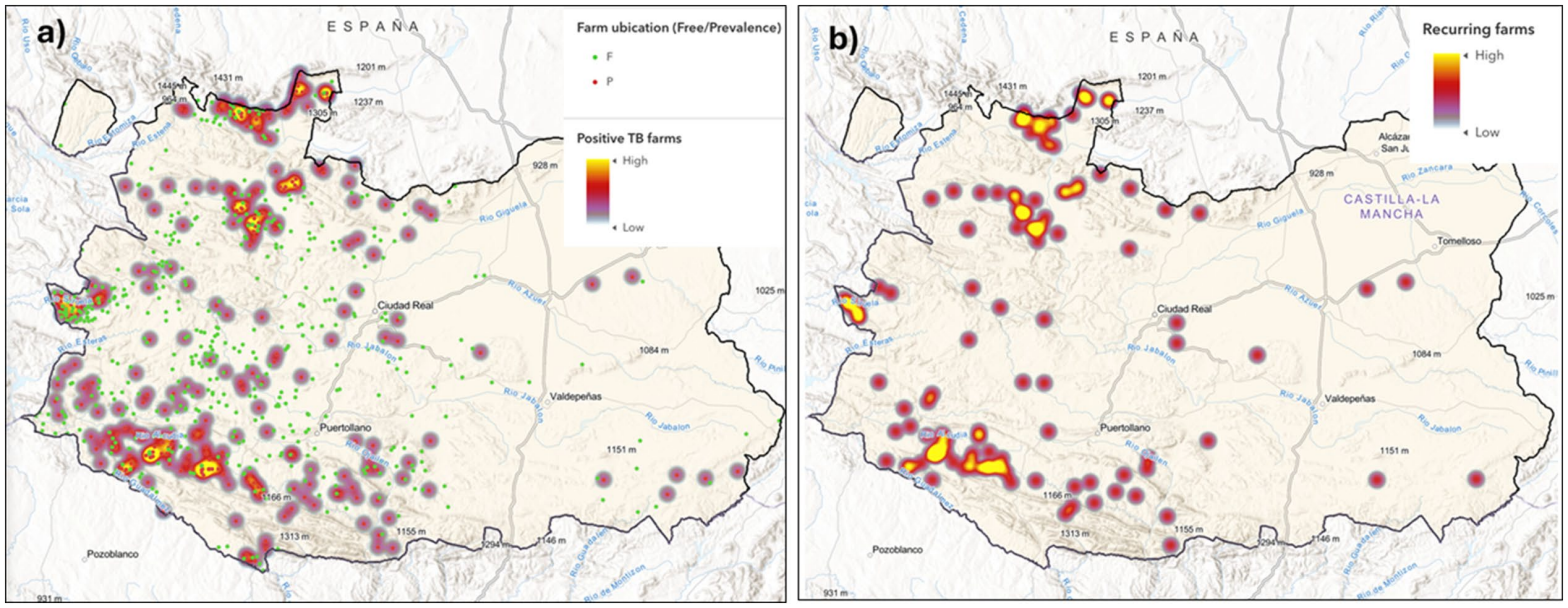
Heatmaps generated using ArcGIS, illustrating the accumulation of bovine farms positive for tuberculosis **(a)**, or the epidemic situation **(b)**—including occurrence, incidence, or recurrence—in Ciudad Real, Spain (2007–2011).

**Figure 9:**
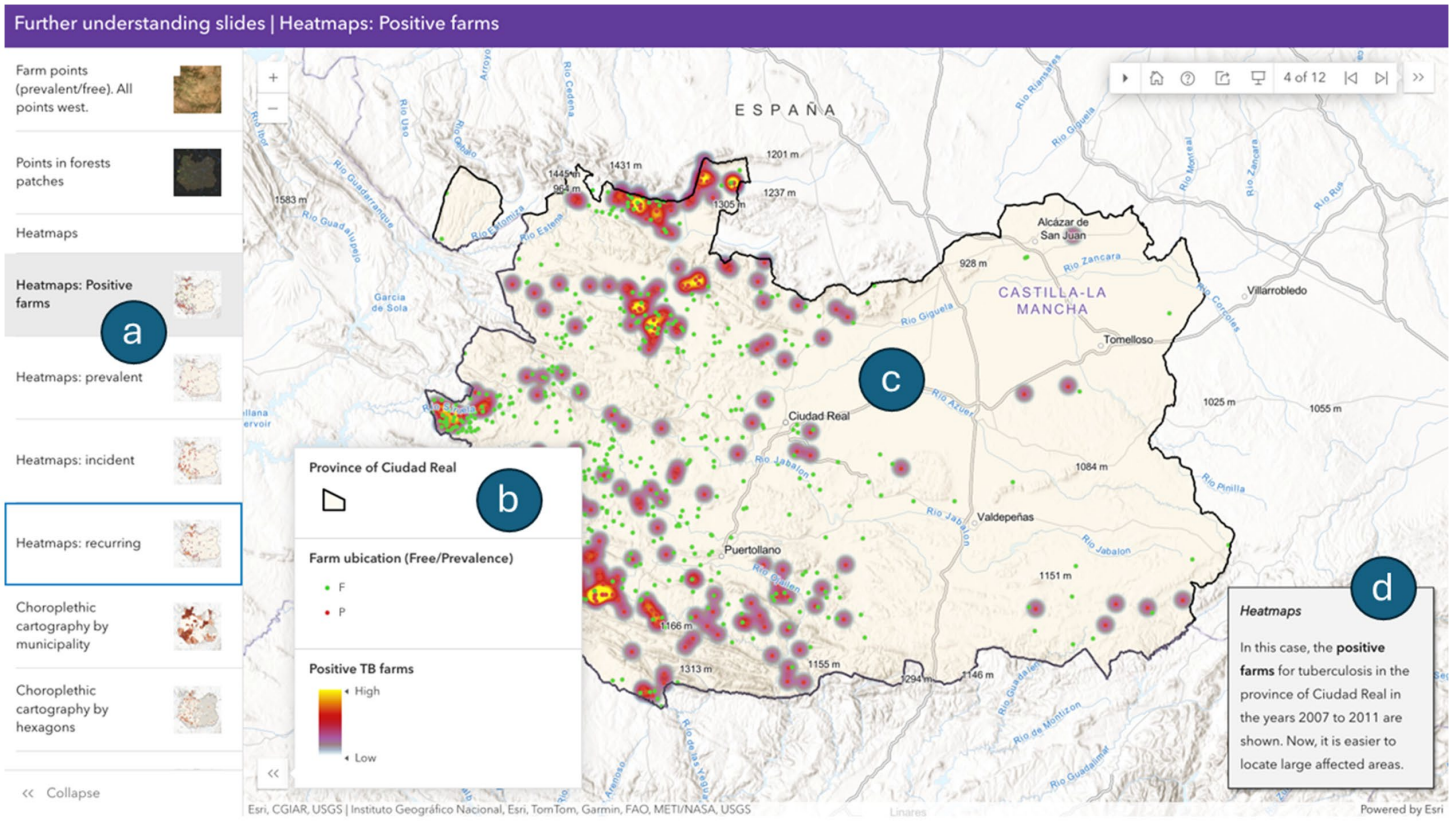
Story map about Animal Tuberculosis in Ciudad Real, Spain (2007–2011) (ArcGIS Online tool). **(a)** Slides with different map cases; **(b)** current map legend; **(c)** typical cartographic result in epidemiological management and research; **(d)** text square with information about the current map slide.

Using ArcGIS Pro, we obtained different heatmaps (Figure 8). We developed a web-based tool to enable students to easily represent the spatial TB patterns and associated risks based on epidemiological data. This tool integrates data from both sides of the interface to allow students to generate diverse spatial projections of epidemiological parameters. The map viewer includes a dynamic legend and a detail panel, along with a text menu (story map) that provides additional information to enhance the understanding of variables, and to support further cartographic and geographic analyses (Figure 9).

## 4 Discussion

Given the existing educational gap in GIS-related competencies among veterinary students, and in medical disciplines in general, the outcomes of this work have led to the development of applied educational resources whose aim is to enhance students’ proficiency in these areas. As mentioned in the Introduction, GIS holds significant value for not only training veterinarians (7, 11, 12), but also for other public health professionals (13) or graduates in other academic fields (8), and even at the pre-university level (9).

To address these educational limitations, we structure this teaching innovation project according to three different complexity levels:

### 4.1 First level: official rabies data in Europe integrated into the European rabies bulletin map

Although the map editing options of the ERB are more limited compared to tools like Epi Map or QGIS, the ERB mapping platform provides a straightforward method for creating epidemiological maps using an extensive database of rabies cases in Europe. The interface allows users to select preferred options for representation in a simple and intuitive manner. One tab is for filtering and selecting rabies cases to display them, while another tab facilitates layer management. This simplicity is particularly advantageous for students with no prior GIS experience. The straightforwardness and limitations of these maps contrast with the significance of the available dataset previously described in the Materials and Methods section. It allows for the filtering and selection of cases in relevant animal species within each group, such as dog, cat or stray dog in domestic animals, or red fox, racoon or other species for wildlife. Bats are considered separately. While the representation of cases lacks precise geographic data (coordinates), it is possible to visualize the location in the province or county (NUTS3) and to visualize recent developments of the rabies situation in Europe, such as the re-emergence in Poland in 2021 (21). The options to represent cases from a single country or to zoom in to a specific country are also available. Any timeframe can be selected to include cases on the layer from 1977 to the current year, which allows the specification of the quarter.

Additionally, this application facilitates the evaluation of oral vaccination campaigns for red foxes, whose aim is rabies control across the continent (22, 23). This is done by selecting the year or years of vaccination to be represented as the area where Oral Rabies Vaccination (ORV) was performed, which is colored on the map. It is possible to include the vaccination areas from 1978 to 2015 to represent one or several years combined by selecting them from a menu. All this information can be exported for educational simulations or epidemiological studies. Although it lacks precise geographic data (coordinates), it is available at the administrative divisions level (up to NUTS3).

Using the data contained in the ERB, we have developed practical exercises with which students create point (symbol) epidemiological maps of the rabies cases reported in any European country since 1977 (Figure 1). These maps include cases involving humans, domestic animals, wild species and bats. We have also incorporated exercises in which veterinary students simulate various rabies epidemiological scenarios across different European countries and evaluate the effectiveness of fox vaccination campaigns using data from rabies prevention efforts in Europe (Figure 2). This documentation has been developed for students at the Faculty of Veterinary Medicine of Murcia, and it is available in the institutional repository of the University of Murcia (Table 3). Although currently available in Spanish, modern translation tools effectively mitigate this limitation for interested users from other language backgrounds. With these exercises, students assess the epidemiological significance of different wild and domestic hosts, along with the unique characteristics of rabies in bats (treated as a separate host group due to specific bat epidemiological features). They also identify the most affected countries and integrate the rabies problem into the One Health framework by considering both human cases and animal hosts. Furthermore, in a straightforward manner, students begin to understand the interaction of different map components and the relation between GIS and epidemiology, i. e., their integration as spatial epidemiology. They gain insight into the importance of various hosts, the most affected European countries, and the impact of fox vaccination on rabies control and the evolution of the rabies situation in Europe. According to our experience, by completing these simple exercises, even students with no prior knowledge of the disease are capable of answering highly specific questions about the complex epidemiology of rabies in Europe.

### 4.2 Second level: use of a simulated database (Coinfame) in Epi map and QGIS

The “Infame” database was created for educational purposes and subsequently adapted for us to work with spatial data by generating the “coinfame_map” dataset. The original dataset refers to a hypothetical disease presented as an epidemic that affects swine farms by causing a reproductive syndrome characterized by infertility, abortions, neonatal mortality and embryonic mortality (Inf-A-M-E), attributed to a fictitious bacterium named *Piglandella infami*. Although this new dataset (“Coinfame”) maintains the reproductive profile of “Infame,” its fictitious nature offers significant advantages for individuals without expertise in infectious diseases. This does away with the need to invest time in defining epidemiological characteristics or other relevant disease features, which is often required for the other two scenarios in this project (rabies or TB).

To support the introduction of GIS to individuals without prior experience, the materials used to develop the project are available, including databases, the necessary vector files, the written manuals or the tutorial videos detailing the exercises (Table 3). Using these resources, students without prior GIS training are guided to employ the Epi Map program, load base layers and data layers, and develop epidemiological maps, including point (symbol) maps, case clustering maps (with a temporal diagram illustrating epidemic progression) and choropleth maps (Figure 3). As mentioned in the previous section, since 2021 we have successfully integrated practical geographical exercises using ERB and EpiMap into the EZPH course. Over this period, we have observed that, thanks to the developed tutorial materials (texts and videos), students highly engage with these tools and effectively solve practical cases using the self-learning materials and the instructor’s role has been limited to addressing specific questions. This allows the session to focus on discussing the epidemiological insights that derive from maps and the unique role of spatial geography as a tool in epidemiology.

Students’ ability to easily learn how to create epidemiological maps using Epi Map is a highly valuable objective. This is due to the importance of spatial epidemiology in professionals’ training across various fields, and particularly in veterinary medicine. According to our experience, students quickly become proficient in using Epi Map to create maps and to subsequently apply it to projects in other courses, or even as for their final degree projects (24). To reinforce the learning of GIS-related methods, we have included the use of various vector file formats, such as kml and shp, at this first level instead of more robust and modern formats like gpkg (which we introduce at the next level). The aim of this approach is for students to become familiar with them, especially with the multifile structure of shp files, and to teach them how to manage different format files in a GIS environment.

Although Epi Map is a relatively simple GIS tool, it is an excellent introduction for beginners. It is also a valuable resource for students before advancing to more complex GIS platforms like QGIS or ArcGIS. This user-friendly tool, coupled with the QGIS interactive application, provides a practical gateway to the complex GIS environment. Unfortunately, the recent announcement by the CDC (25) regarding the end of technical support and development for Epi Info as of September 30, 2025, introduces uncertainty about the future of this valuable tool.

### 4.3 Third level: use animal TB data in QGIS and Arcgis

This case exemplifies the increase in the quality of the data collected from monitoring and surveillance programs for infectious diseases, as well as their populations, management and environments. The fauna-livestock interface, particularly in Mediterranean environments in the center and to the south of the Iberian Peninsula, generates very complex and dynamic systems, where the identification of the determinants of the maintenance and spread of pathogens, such as the agents causing animal TB, has been the core of several studies (26–28).

The project developed with the TB database, obtained from livestock health campaigns conducted in CR, is more complex than the previous one. It is designed as a task for students with prior GIS knowledge. As a result of the randomization applied to the original distribution of bovine farms, the project does not accurately reflect the real geographical profile of TB in the CR province, but provides a functional dataset (with coordinates) for other exercises and simulations. It also facilitates visual comparisons of the epidemiological profile of TB in extensive farming systems, which are closely tied to the territory and biodiversity. Unlike the previous section, the use of real TB data from livestock health campaigns in an extensive farming context not only enhances GIS training but also provides students with hands-on experience in the epidemiology of a zoonotic disease strongly influenced by wildlife reservoirs, particularly deer and wild boar (29, 30). This significantly enriches students’ educational experience. In Spain, analyses to explain the incidence and persistence of TB in cattle in the CR province showed relations between prevalence in cattle and wild animals’ health status in 2007 (28). In a subsequent analysis for which a longer time series (2007–2011) was applied, and working in this case on farms without aggregating data, it was demonstrated that the TB risk (prevalence, incidence and/or persistence) on farms was related to the disease persistence in previous years, with high livestock censuses and extensive production systems (27). In addition, the presence of intensively managed big game reserves was also related to a higher risk of incidence and persistence in domestic livestock. Once again on at the regional scale of CR, a study was carried out to assess the importance of wildlife and its management in the risk of infection in cattle in extensive livestock farming and to produce risk maps (26). The spatial models revealed the relation between the environmental variables, which explain where the interface takes place, and the farm risk, as well as an association with TB prevalence in wild boars. In short, exploring new (integrated) strategies for controlling animal TB in multihost systems requires clear spatial knowledge of the epidemiological situations that are being addressed.

In relation to the above, we also added the cartography of hunting data by municipalities and hexagons for further analyses. The main represented variable is the total number of wild ungulates harvested during the same period and in the same study area (CR province) as the data recorded on farms by square kilometers (Harv_km^2^). Finally, these maps were included because the exercise of linking wild ungulate abundance data with health data from previous farms provides students with a more comprehensive view of the health status in the study area. We are dealing with an area of high contact between wild game and domestic species, specifically dehesa areas, where knowledge of the One Health concept is a priority.

Another innovation in this section is that, in order to minimize any bias associated with the use of municipal polygons, we developed a shapefile for the CR province featuring a regular hexagonal grid with cells of 20 km^2^. This is a method employed in animal health research within the One Health framework (2). This approach ensures ore uniform spatial representation by reducing the potential biases inherent to administrative boundaries.

It also allows students to reflect on the interpretative biases introduced by the varying polygon sizes (municipalities in this case) used to design choropleth maps. This approach enables students to assess different visualizations by presenting health information through irregular municipal polygons, regular hexagons and raster maps of point density (heatmap), and facilitates the detection of changes in the distribution patterns of disease and risks (Figures 6–8). The use of administrative units (municipality polygons) is more accessible and practical for management and decision making, but is less homogeneous and has lower spatial resolution in a pattern analysis. Therefore, the use of a hexagonal grid (20 km^2^/hexagon) offers advantages for being more precise for spatial detail and in-depth research, but is less informative in the administrative context.

The complexity of this database is higher than the previous one (coinfame), and it is primarily designed for developing projects in QGIS or ArcGIS rather than in Epi Map. However, as the repository includes both databases and the municipal or hexagonal polygons, epidemiological maps can also be created using Epi Map. This approach is like the previous phase and is well-suited for students with limited GIS knowledge because the software is easy to use. In addition to creating maps, the database provided in this objective allows students to engage in other exercises during practical epidemiology sessions, such as solving problems related to health indicators and other analytical tasks. In this context, students work with real-world data rather than with simulations, which enables instructors to expand on the epidemiological aspects of TB in much more detail.

The dataset included in the final interactive project, including fine-scale mapping, allows students to pinpoint the specific locations where wildlife and livestock interact, and to identify hotspots of disease risk for TB transmission. By managing appropriate GIS tools, they learn that precise mapping is essential for implementing effective disease control measures, such as movement restrictions or interface management. The wildlife-livestock interface is a complex ecological system, and fine-scale mapping helps to understand species interactions and their influence on disease transmission. Furthermore, students recognize that mapping disease on this scale requires a combination of traditional epidemiological tools and cutting-edge technologies. For these purposes, GIS are fundamental for spatial data visualization and analyses, and can be complemented by other approaches that also require spatial techniques:

(i) Remote sensing (these data can be used to assess habitat suitability for different animal species and to monitor changes in the environment that may influence disease risk)
(ii) GPS Tracking and Telemetry (GPS collars and other tracking devices allow researchers to monitor individual animals’ movements at the interface) for understanding animal interactions and identifying potential pathways for disease transmission
(iii) Molecular Epidemiology Tools, such as PCR and genetic sequencing, are used to identify and characterize pathogens to trace the origins of disease outbreaks
(iv) Epidemiological Modeling. It is used to simulate disease transmission dynamics, predict the spread of outbreaks, and evaluate the effectiveness of different control measures

This case study aims to easily represent the spatial patterns of disease (TB) and risks based on the epidemiological information obtained at high resolution on a large scale (CR) and to promote students’ understanding of the possibilities of spatial projections of epidemiological parameters, which would later require deeper learning about GIS tools.

Students are introduced to the potential of combining these tools and resources, to gain a more comprehensive understanding of disease dynamics at the wildlife/livestock interface, and to develop effective strategies for disease prevention and control. As previously mentioned in the sections above, the perceived improvement in students’ GIS-related competencies and engagement is currently based on instructors’ observations, derived from direct interaction with students and the evaluation of their submitted exercises. However, a standardized survey is planned for future implementations of the course, targeting students exposed to the three different levels of complexity developed in this project. This will allow for a more objective assessment of student progress and will help identify opportunities for further refinement of the educational approach.

### 4.4 Integrating our results into an open-access environment

All the data and materials generated through this project are openly accessible, not only to the students at our universities, but also to anyone interested (Table 3). These include the generated databases, as well as tutorials for developing exercises, available in both written and visual formats using the Epi Map and the ERB mapping options. By leveraging open platforms like the institutional repositories, the University of Murcia TV channel or QGIS or ArcGIS interactive online tools, these resources go beyond the institution where they were created. In addition, the theoretical introductory content on GIS, along with self-learning exercises adapted for Spanish veterinary students, is also available in Table 3. This allows external users to engage in self-directed learning in geographic epidemiology applications. This is particularly significant because user guide Epi Map videos for post-Epi Info™ 7 versions are lacking (31), tutorial videos for Epi Map are scarce (33) and existing materials are complex for individuals without prior GIS knowledge. Moreover, these open-access resources have the additional advantage of being cost-free, which facilitates broader GIS training opportunities in educational settings with limited financial resources.

The QGIS project based on the dataset of a fictitious rabbit disease (coinfame) is available as an interactive html file (Table 3). In the initial project development phase, we used the QGISCloud application to upload a demonstration project to allow students to compare their results. However, this option became limited due to the inclusion of raster layers (i.e., the heatmap), which increased the project size beyond the 50 MB limit of the QGISCloud free server. Consequently, and given that the use of open-access, free tools, is a priority in this project, we opted to reduce the demonstration projects to the options offered by QGIS through the QGIS2web plugin OpenLayers in html format files (Figure 4) and by publishing the online map throughout QGISCloud without the heatmap layer (Figure 5).

Although QGIS allows the addition of descriptive information to its projects to create basic story maps, its limited functionality in this regard led us to use the ArcGIS platform instead. Although ArcGIS is paid software, its online publishing option enables the final project to be made available to students via an open-access link. This functionality allows the incorporation of explanatory texts, which enable students to integrate essential information about the disease and key interpretations of data visualizations. Story maps have proven to be a highly valuable educational resource that is well-received by students. In fact, it has been concluded that there is a critical need for digital narration as a teaching and learning method in 21st-century education in the geography field (10), a conclusion that could be extended to GIS education in other fields, including veterinary studies.

Students in initial GIS training stages can compare their results using the coinfame dataset in Epi Map with the interactive map presented as an html file and online QGISCloud publication. These include similar functionalities, such as choropleth maps, case maps and cluster maps. They can also explore more advanced features created in QGIS that are unavailable in Epi Map, such as heatmaps and adding alternative basemap options like Google Satellite or Positron without labels. Furthermore, students with a more advanced GIS competence level can utilize these databases to initiate projects in QGIS with the data from the interactive map project as a model. Finally, the complete TB project in CR, available as an online story map in ArcGIS, allows users with no prior GIS experience to easily explore different layers, visualize various health situations by geographic location and overlay layers with risk factors, such as wild ungulate abundance. This enables users to become familiar with GIS tools while also gaining insights into TB epidemiology through the information provided on the story map.

Finally, these educational materials, particularly those related to the TB dataset, can be applied in other educational contexts, such as postgraduate or master’s programs. They provide the foundation for more advanced projects in QGIS, including geoprocessing and other analyses relevant to GIS training. Table 1 summarizes the strengths, limitations and challenges of using these GIS applications for educational purposes, along with the main educational objectives to be fulfilled. Beyond the specific GIS competencies that veterinary students can acquire through these materials, GIS training also offers broader educational benefits. These include fostering independent thinking, enhancing problem-solving skills, promoting learning through trial and error and encouraging creativity in developing solutions, as emphasized in the geography education context (32).

## 5 Conclusion

The use of GIS tools is a necessity for veterinary students, and it is essential to strengthen this learning during university training. Starting with less complex exercises, we propose a series of training activities so that users with no prior GIS experience can easily apply these tools to gain knowledge about the epidemiology of both fictional and real diseases, such as rabies or animal TB. This includes providing users with both tutorials and training documents, as well as the necessary databases, to complete projects, or directly providing access to already developed complex projects online, such as story maps or through interactive files. This will allow students or interested individuals to make sanitary maps with a fictious disease, to visualize the rabies situation in Europe or to pinpoint specific locations where wildlife and livestock interact, and to identify hotspots of disease risk for animal TB. By managing appropriate GIS tools, they will learn that precise mapping is essential for implementing effective disease control measures, such as movement restrictions or interface management.

## Data Availability

The original contributions presented in the study are included in the article/supplementary material, further inquiries can be directed to the corresponding author.
